# Detection of *Clostridium perfringens* in donor milk at a human breast milk bank: a case report

**DOI:** 10.1186/s12879-023-08822-8

**Published:** 2023-11-17

**Authors:** Ke Liu, Zhiyong Liu, Xufei Chen, Rouzhi Wang, Dan Wang

**Affiliations:** 1 Department of Obstetrics and Gynecology, the First Affiliated Hospital of the Army Medical University, Chongqing, 400000 China; 2 Clinical Laboratory, the First Affiliated Hospital of the Army Medical University, Chongqing, 400000 China

**Keywords:** *Clostridium perfringens*, Donor human milk, Breast milk bank, Microbiological testing

## Abstract

**Background:**

The breast milk bank is a professional organization that collects donor human milk (DHM) for special medical needs by recruiting qualified breast milk donors. Such organizations are also responsible for the disinfection, processing, testing, storage, distribution, and use of breast milk. As DHM is a biological product, it may get contaminated. Microbiological testing is the final step to determine microbial contamination of DHM. However, a universal method for the microbiological analysis of DHM in breast milk banks globally is lacking.DHM without strict screening may become a potential carrier of pathogens and seriously threaten the health of infants. *Clostridium perfringens*, a gram-positive anaerobic bacterium, is capable of causing wound infections, including gas gangrene, enteritis/enterocolitis, and enterotoxemia. Here, the first case of *C. perfringens* detected in DHM has been reported to facilitate the identification of such contamination in breast milk banks.

**Case presentation:**

A breastfeeding mother donated 3000 mL of milk to the breast milk bank of the First Affiliated Hospital of the Army Medical University(over 2900 beds and patient receiving capacity of over 132,000), Chongqing, China. The milk sample was subjected to microbiological screening using liquid enrichment, followed by anaerobic and aerobic culturing. The results revealed the growth of *C. perfringens* in the anaerobic culture medium, but no bacteria or yeast-like fungi were observed in the aerobic culture medium. The donor did not exhibit any clinical symptoms, and her routine blood results and body temperature were normal. However, the infant fed with her milk had recurrent bloody stools. Breast milk bank infection control emergency handling as well as environmental sampling and investigation revealed that the cause was contamination of the donor’s home-use breast pump with *C. perfringens*. The infant no longer experienced bloody stool once the donor changed the breast pump.

**Conclusions:**

*C. perfringens* can enter breast milk from contaminated pumping environments or devices, thus causing illness in infants. The microbiological testing of DHM in breast milk banks can be accomplished using liquid enrichment, along with anaerobic and aerobic culture, which is of immense significance in improving the standards for microbiological screening, DHM safety, and infant health.

## Introduction

*Clostridium perfringens* is an opportunistic pathogen capable of causing wound infections such as gas gangrene (spindle-like muscle necrosis), enteritis/enterocolitis (the most common human foodborne disease), and enterotoxemia [1]. C-type and F-type strains can cause hemorrhagic necrotizing enteritis in neonates, as well as food poisoning and diarrhea. The foods contaminated by *C. perfringens* that cause foodborne diseases are mostly protein-rich meat and milk. Breast milk is rich in nutrients, such as proteins, fats, and carbohydrates, and contains a complex and diverse microbial community. When the storage environment is unclean, or the storage temperature is inappropriate, breast milk can become a good culture medium for bacteria. Utilization and sharing of DHM contaminated with bacteria and fungi may lead to the spread of pathogens [2], thereby threatening the health of infants. A case of contamination of donor human milk (DHM) in a breast milk bank has been reported herein. Liquid enrichment, followed by anaerobic and aerobic culture, was used to detect *C. perfringens* in DHM.

## Case presentation

The donor lives in the first-class city of Chongqing in China. On October 19, 2020, the donor underwent a cesarean section in the obstetrics department of the First Affiliated Hospital of the Army Medical University(Chongqing, China). The newborn baby boy weighed 3400 g. She did not face any pregnancy-related complications. The surgery was smooth, and the postoperative recovery was good. On December 8, 2020, the breastfeeding mother donated 3000 mL of frozen breast milk to the breast milk bank of the hospital mentioned above. The donor signed the consent for free donation of breast milk. The donor had no history of smoking, drinking, drug abuse, long-term treatment with medications, blood product infusion, or food/drug allergies in the past 6 months. Serological test results for HIV, hepatitis B, hepatitis C, syphilis, and cytomegalovirus were negative, and nucleic acid test results for SARS-CoV-2 were also negative. Body temperature, pulse rate, and blood pressure were normal. The breasts were full, and there were no hard lumps on palpation. Milky white milk flowed out when pressed, and there was no tenderness. The nipple and areola were undamaged.

The home breast milk pump purchased by the donor was used for collecting milk, and the device at that time was 2 months old. The donated DHM storage container was a breast milk storage bag that was placed in a household independent refrigerator and frozen in a drawer of a − 16℃ independent refrigeration room. There was no obvious dirt or odor inside the refrigerator.DHM samples in the batch were all frozen breast milk collected within 2 months after delivery and stored for < 30 days. The outer packaging of the DHM was complete, without any damage or air leakage, according to the requirements of the Chinese National Standard GB5408.1-1999 for pasteurized milk. The color, odor, and tissue state were normal. The color was uniform and milky white, without any odor. After thawing, no precipitation, clumping, or stickiness was observed, and the tissue state was uniform and liquid-like.

**Fig. 1:**
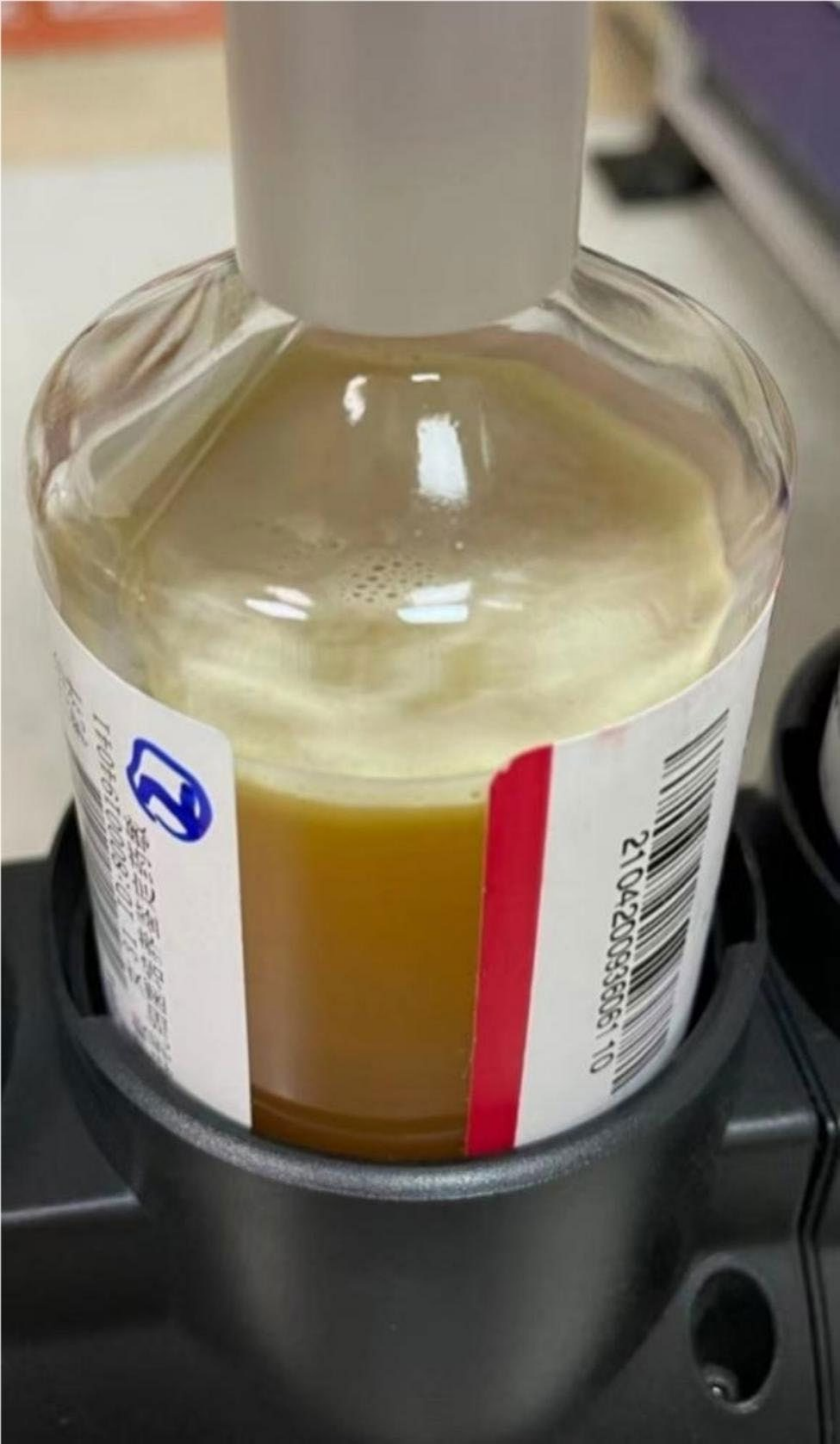
DHM-enriched liquid culture medium

On December 8, 2020, The breast milk bank warehouse conducted microbiological testing on this batch of DHM. As all DHM samples must undergo routine aerobic and anaerobic bacterial culture analysis before storage, the breast milk bank thawed, mixed, packaged, and pasteurized this batch of DHM, and retained the milk samples before and after pasteurization for bacterial aerobic and anaerobic culturing and bacterial identification routinely to perform microbiological screening before storage. According to *The Establishment and Management of Human Milk Bank in Chinese Medical Institutions*(T/CNSS2020-003), DHM bacterial test results after pasteurization showed no bacterial growth. The samples were collected, and the bacteria were isolated and cultured according to the Chinese Standard Procedure for Clinical Microbiological Examination (ISO15189); the mixed milk samples were taken and injected into anaerobic and aerobic culture media. The aerobic and anaerobic culture medium used was Thermo Scientific liquid microbial culture medium (Rui Enzyme Company) and was incubated at 35℃–37℃ for 120 h. Thermo Scientific’s fully automatic rapid microbiological and biochemical identification instrument was used to identify the bacterial species in the samples (Fig. 1).

**Fig. 2:**
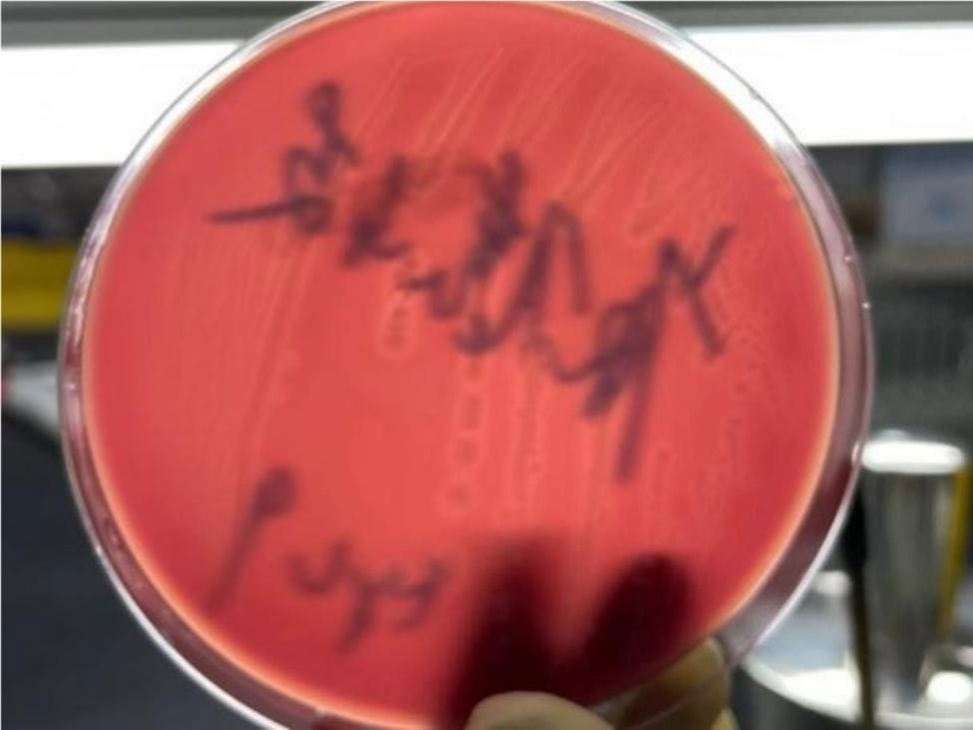
*C. perfringens* colony morphology after anaerobic culture(Columbiabloodagar base medium)

**Fig. 3:**
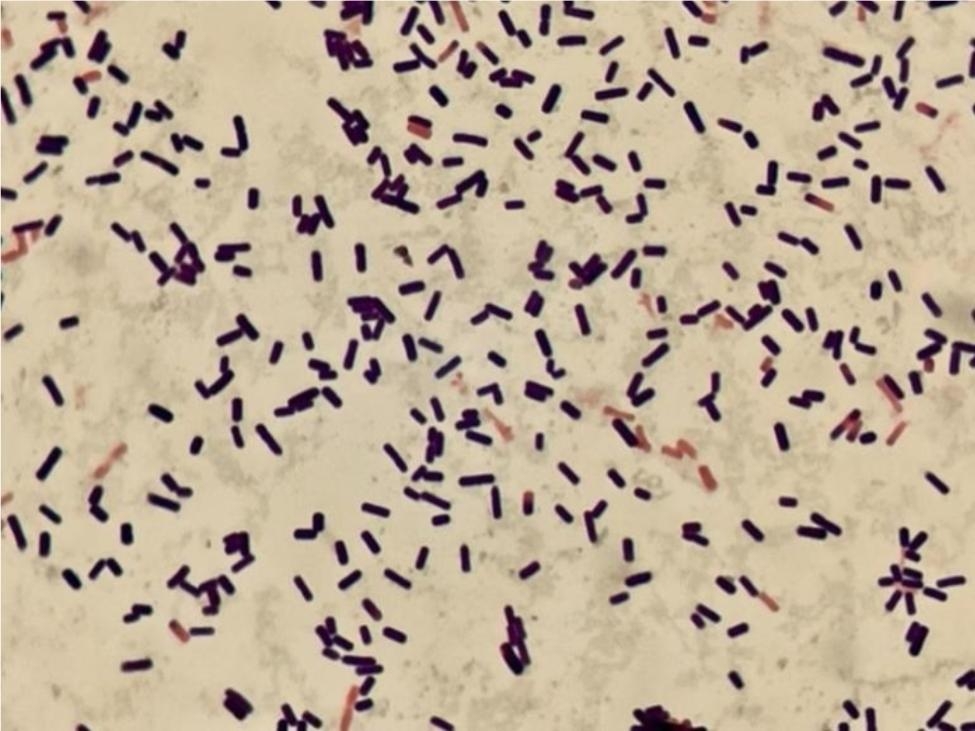
Microscopic morphology of *C. perfringens* after anaerobic culture

At 11:36 AM, December 11, 2020, the microbiological laboratory reported a critical finding that *C. perfringens* was detected in breast milk both before (Fig. 2) and after pasteurization (Fig. 3). At midnight, the emergency response program of the breast milk bank was launched. Owing to the lack of DHM distribution within 1 week, the recall process was not initiated. The hospital’s infection control department sealed the warehouse of the breast milk bank and conducted emergency sampling on the interior of the refrigerator, refrigerator door handles, ultra-clean workbench tops, the interior of the pasteurization water bath, and the hands of the staff members. Furthermore, DHM from other mothers in the refrigerator were randomly selected for sampling and culturing. After the sampling swab was fully eluted in the sample solution, aspirate eluent and injected into the liquid culture medium for aerobic and anaerobic cultivation of C. perfringens, and then aspirate 1.0ml of eluent again and inoculated into a petri dish, and 15–20 mL of nutrient agar medium (cooled to 40–45℃) was poured into each petri dish, followed by culturing in a 36 ± 1℃incubator for 48 h and enumeration of colony count. The causative organisms were isolated if necessary. Five days later, on December 16, 2020, the sensory control department reported that *C. perfringens* was not detected in the sampling results of the breast milk bank. The findings are listed in Table 1.

**Table 1:** Environmental hygiene-testing report for an emergency sampling of breast milk bank

Item	Test specimen	Result	Reference range
Hand hygiene	Hygienic hands of operators at the breast milk bank	3.33 cfu/cm^2^	≤ 10 cfu/cm^2^
Object surface	-20°C refrigerator door handles	1.4 cfu/cm^2^	Class II area II: ≤ 5 cfu/cm^2^
	-20°C refrigerator drawer	0.2 cfu/cm^2^	
	The interior of a 4°C refrigerator	2.3 cfu/cm^2^	
	Ultra-clean workbench tops	1.1 cfu/cm^2^	
	The interior of the pasteurization water bath	0 cfu/cm^2^	
Breast milk samples	Donor1’s Breast milk 1 (before pasteurization)	*S. epidermidis*	
	Donor2’s Breastmilk 2 (before pasteurization)	*S. epidermidis*	
	Donor3’s Breastmilk 3 (before pasteurization)	*S. vestibularis*	
	Donor3’s Breastmilk 3 (after pasteurization)	No bacteria or yeast-like fungi	

On December 12, the breast milk bank notified the results of the donor milk microbiological analysis and followed up on the health status of the infant. This infant was exclusively fed with breast milk, breastfeeding during the day and bottle feeding using the breast pump at night. On November 19, the infant experienced the first appearance of blood-containing feces, which improved after being fed with amino acid formula (AAF). Later, the infant was fed breast milk and AAF, and the symptom of bloody stools reappeared. On December 2, blood-containing feces and vomiting, accompanied by runny nose and coughing, were observed, and medical attention from the pediatric department of an external hospital was sought. The feces routine test showed 1–3 red blood cells per high-power field (reference value: negative). The diagnosis was blood in the stool(ICD: K92.201), possibly caused by intestinal milk protein allergy, tracheitis, or rhinallergosis. The results are presented in Table 2. Cow milk protein allergy (CMPA) may lead to bloody stools, and common symptoms include bloody stools, bloating, and diarrhea [3]. The treatment involved discontinuing breastfeeding, probiotics supplementation, and switching to AAF feeding, and the mother avoided milk protein-containing foods for 1 week and then switched to AAF + breastfeeding on December 9. On December 12, the symptom of bloody stools appeared again. Based on the poor efficacy of AAF + food avoidance therapy and the results of the bacterial culture of the milk sample, CMPA was ruled out. Milk contamination by C. perfringens was considered, and breastfeeding was discontinued again. On December 18, 2020, the donor visited the outpatient clinic for treatment and adopted hand milking to collect fresh milk on-site for microbiological analysis. On December 22, the milk culture results showed that *S. epidermidis* was grown in the aerobic culture and that there was no growth of bacteria or yeast-like fungi in the anaerobic culture. This finding eliminated the presence of *C. perfringens* in the milk sample; hence, contamination from breast suction tools or environmental pollution was considered.

**Table 2:** Infants’ routine feces testing results

Item	Results	Reference value	Unit
Color and appearance of feces	Yellow, Paste		
Red blood cells (feces)	1–3	Negative	/HP
White blood cells (feces)	0–2	0–3	/HP
Phagocyte	Negative (-)	Negative	
Egg	Negative (-)	Negative	/LP
Occult blood test (monoclonal)	Negative (+)	Negative	
Reducing sugar test	Negative (-)	Negative	

On December 14, 2020, the donor collected fresh milk using a breast pump at home 6 days after the breast milk donation and sent it to our laboratory for repeat microbiological testing. On December 16, *C. perfringens* was detected in the anaerobic culture medium and *Staphylococcus epidermidis* in the anaerobic culture medium.

On December 23, 2020, the staff of the breast milk bank visited the donor’s home for environmental inspection and sampling and submitted samples from the interior of the breast milk storage refrigerator, refrigerator door handles, breast pump interior, donor hand, desktop, and community soil samples. On December 28, the sampling results revealed the presence of *C. perfringens* in the breast pump and the community soil. The organism was not detected in any other samples. Table 3 summarizes the process of investigating the source of infection.

**Table 3:** Investigation of the source of infection

Date	Incident	Results
December 14, the breast milk donation	Re-inspection: Collection of the milk using a home breast pump and sending it for microbiological analysis	Anaerobic culture: *C. perfringens* at the production stage Aerobic culture: *S. epidermidis*
December 16, the breast milk donation	Follow-up on the infant’s health status	According to the return visit, Symptoms of bloody stools persisted between November 19 and December 11, and there was no improvement even after the mother avoided milk protein food and AAF feeding. CMPA diagnosis was ruled out, and milk contamination was considered. Breastfeeding was discontinued.
December 18, the breast milk donation	Second retest: Hand milking at the outpatient clinic to collect fresh milk and send it for microbiological analysis	Anaerobic culture: no bacteria or yeast-like fungi Aerobic culture: *S. epidermidis*
December 23the breast milk donation	Sampling and detection of donor’s home environment	Breast pump internal: *C. perfringens* was detected Community soil: *C. perfringens* was detected Breast milk storage inside a refrigerator, at the refrigerator door handle, mother’s hand hygiene, and desktop sampling did not detect *C. perfringens* at the production stage

### Outcome

The breast milk bank environment utilized chlorine-containing preparations to wipe the surfaces of refrigerators,ultra-clean workbenchs, and other objects, and the air was finally disinfected with hydrogen peroxide. After passing environmental hygiene testing, the warehouse was reopened, and *C. perfringens* was no longer detected. After replacing the breast pump by the donor, the donor first wiped the refrigerator and other objects surfaces at home with chlorine-containing preparations and then disinfected the bottle and the bottle brush with hypochlorite and dried them completely, followed by resuming breastfeeding. The baby’s bloody stool symptoms completely disappeared after this, and hence, breastfeeding was continued until the infant was 8 months old.

## Discussion

Being a mild anaerobic Gram-positive bacterium that can form spores, *C. perfringens* is widely present in soil and natural water. This organism is a part of the normal gut microbiota in humans and animals [4]. The bacterium can produce approximately 20 potent toxins, which can be classified into subtypes A–G7 according to the toxin production patterns of various strains [5]. The resulting diseases include muscle necrosis (gas gangrene), food poisoning, necrotizing enteritis, necrotizing enterocolitis, and bacteremia. The C-type strain can cause necrotizing enteritis and enterotoxemia, and neonates are particularly susceptible [6]. The clinical manifestations are diarrhea and abdominal pain, and the macroscopic and microscopic pathological features are necrotizing enteritis and enterocolitis [7, 8], which are consistent with the symptoms of infant diarrhea and bloody stool in this case. *C. perfringens* can cause necrotizing enterocolitis (CPA-NEC) associated with *C. perfringens*. NEC is the most serious and fatal neonatal gastrointestinal emergency reported worldwide. It is an acquired inflammatory bowel disease characterized by tissue necrosis in the gastrointestinal tract. Extremely low birth weight (< 1500 g) occurs in 5–15% of all preterm infants, resulting in high mortality (approximately 40%) and serious long-term complications [9]. Especially for premature and low-birth-weight infants, the intake of breast milk contaminated by *C. perfringens* may lead to serious consequences, such as necrotizing enteritis and enterotoxemia, owing to the unstable establishment of gut microbiota and immature intestinal development.

Breast milk is the preferred choice for the enteral nutrition of neonates and is the ideal natural food for infants and young children. However, the rich nutritional components in breast milk are also conducive to the growth of bacteria. It has been demonstrated that [10] the bacteria in breast milk reproduce in the intestines of infants, which significantly impacts the development of gut microbiota. Breast milk can be contaminated by bacteria in various stages, from collection to consumption, and can become a carrier of symbiotic as well as pathogenic bacteria either from mothers or from the environment. The collected breast milk samples showed bacterial growth over 75%, and 7–36% contained pathogenic bacteria [11]. This finding may be related to the mother’s hand hygiene and the cleaning of breast suction tools. Breastfeeding mothers often use breast pumps for milk collection. Before breast milk was collected from the donors, proper breast milk collecting guidance was provided to them. However, owing to the complex internal structure of breast pumps, disassembling and cleaning them is difficult, and the humid internal environment may aid the growth of harmful bacteria. Breast milk collected using the breast pump is highly susceptible to contamination. In the case reported in this article, *C. perfringens* was cultured in samples obtained from the internal environment of the donor’s home breast pump and in soil samples from residential communities. Therefore, it was speculated that the bacteria in the soil environment contaminated the breast pump and that the mother’s inadequate hand hygiene and incomplete cleaning of the breast pump resulted in *C. perfringens* contaminating the collected breast milk. Hence, the mother must ensure that she thoroughly cleans her hands before breastfeeding. When using a home/hospital breast pump, it is necessary to pay careful attention to cleansing and disinfecting. After breast pumping, breast pumping accessories should be washed and bleached with a detergent, followed by sterilization with atmospheric steam in a bottle sterilizer pot (independent electrical or microwave oven sterilizer box or sterilizer bag), and finally dried and stored in a dry environment [12].

The report was the first case reported of *C. perfringens* detected in DHM worldwide. Pasteurization cannot eliminate *C. perfringens* in DHM as the organism can produce highly resistant spores that survive even after pasteurization and get converted to vegetative forms when exposed to nutrients in the human body under appropriate conditions. In this case, the DHM of the mother was stored in a separate refrigerator at − 18 °C. In a low-temperature environment, the spores produced by *C. perfringens* remain in a dormant state. When pasteurizing the DHM, the spores germinate, grow, reproduce rapidly, and produce enterotoxin under the stimulation of a 62.5 °C constant temperature water bath. The spores produced by *C. perfringens* exhibit strong thermal resistance, and D100 (the time required to reduce spore activity by 1 log at 100℃) of the spores ranges from ≥ 30 to 120 min [13]. In this case, *C. perfringens was* detected in DHM even after pasteurization. Therefore, pasteurization is an effective method to ensure the microbial safety of DHM in the breast milk bank. Microbiological testing can verify and screen the effectiveness of pasteurization, which is the final key step in confirming the effect of sterilization. In the Perron Rotary Express breast milk bank in Australia [14], strict microbiological testing was performed on DHM after pasteurization, and 26% of DHMs were discarded because of non-compliance. *C. perfringens* is an anaerobic bacterium, and its microbiological testing requires the use of an anaerobic culture [15]. In this case, the microbiological testing results of DHM showed that the aerobic culture result was negative, whereas the anaerobic culture result was positive for *C. perfringens*. If anaerobic culture and identification are not conducted, false negative results would likely be obtained, thus leading to the ingestion of contaminated DHM by infants and serious consequences. Since the establishment of the breast milk bank at our hospital in 2015, DHM requires routine aerobic + anaerobic bacterial culture and bacterial identification before and after disinfection, and the DHM after disinfection can only be distributed and used without any bacterial growth. According to the current database, 1691 DHM samples were examined in 8 years, of which bacteria colonized 47%, 22.1% contained pathogenic bacteria even after pasteurization, and the isolated pathogens included two types of anaerobic bacteria, *C. perfringens* and *Clostridium tertium*, as well as fastidious bacteria such as *Nocardia* were detected. Hence, in breast milk banks, microbiological screening of DHM should consider the possible contamination by anaerobic and fastidious bacteria and adopt stringent methods for microbiological analysis after pasteurization. Liquid enrichment culture can be used, and necessary anaerobic and aerobic cultures can be performed to improve the bacterial detection rate.

Pasteurized DHM is a suboptimal choice for neonates, especially for premature babies and infants with low birth weight who cannot consume fresh mother’s milk [16]. The case reported herein suggests that there may be anaerobic bacteria in DHM, and pasteurization may not be able to kill all pathogenic bacteria and their spores. Therefore, a hazard analysis and critical control points system should be established in breast milk banks to assess the risks associated with DHM processing. The results of this case presentation confirm that strict microbiological testing of DHM after pasteurization is one of the critical control points for the operation of breast milk banks. Such testing can prevent, eliminate, or reduce the potential safety hazards of DHM from collection to consumption and ensure the quality and safety of the breast milk bank operation. At present, there are no unified standards for the microbiological testing of DHM in breast milk banks globally. The process mostly relies on blood agar plates for cultivation [17]. The sampling methods used for detecting contamination of DHM include random, systematic, and stratified sampling. The methods used by breast milk banks in different regions also vary. There is a lack of research on the microbiological analysis of DHM in breast milk banks. In the case reported here, the same batch of DHM from the same donor was mixed and sampled, and a liquid enrichment culture was used for microbiological testing. Two samples were selected for both anaerobic and aerobic cultures, which improved the bacterial detection rate. However, long testing times and high testing costs are some of the shortcomings. Finding faster, more accurate, more convenient, and more effective testing methods is a critical direction in refining the microbiological analysis of DHM.

## Conclusion

This article has reported for the first time the detection of *C. perfringens* in DHM and is an important reference for the safety management of DHM in breast milk banks. This work has shown that the anaerobic bacterium *C. perfringens* present in the DHM cannot be completely eradicated with pasteurization. Based on the findings, it could be suggested that the quality management and infection control of breast milk banks should pay attention to the contamination of DHM by anaerobic fastidious bacteria. Strict microbiological testing of DHM using the liquid enrichment culture method is required. Performing anaerobic + aerobic culture if necessary, strict bacterial screening, and improving the standards of microbiological screening are crucial for DHM safety and infant health. This case report has deepened our understanding of microbiological testing and quality control of DHM in breast milk banks. Furthermore, this study has paved the way for improving the industry standards and guidelines for the operation of breast milk banks to ensure their quality and safety management.

## Data Availability

The datasets used and/or analyzed during the current study are available from the corresponding author on reasonable request.
